# Raman spectroscopy for accurately characterizing biomolecular changes in androgen‐independent prostate cancer cells

**DOI:** 10.1002/jbio.201700166

**Published:** 2017-11-23

**Authors:** Stella Corsetti, Thomas Rabl, David McGloin, Ghulam Nabi

**Affiliations:** ^1^ SUPA, School of Science and Engineering University of Dundee Dundee Scotland; ^2^ Drug Discovery Unit, College of Life Sciences University of Dundee Dundee Scotland; ^3^ Division of Cancer Research, School of Medicine University of Dundee Scotland

**Keywords:** castrate resistant prostate cancer (CRPC), L‐arginine, metastatic prostate cancer cells, phenylalanine, Raman spectroscopy

## Abstract

Metastatic prostate cancer resistant to hormonal manipulation is considered the advanced stage of the disease and leads to most cancer‐related mortality. With new research focusing on modulating cancer growth, it is essential to understand the biochemical changes in cells that can then be exploited for drug discovery and for improving responsiveness to treatment. Raman spectroscopy has a high chemical specificity and can be used to detect and quantify molecular changes at the cellular level. Collection of large data sets generated from biological samples can be employed to form discriminatory algorithms for detection of subtle and early changes in cancer cells. The present study describes Raman finger printing of normal and metastatic hormone‐resistant prostate cancer cells including analyses with principal component analysis and linear discrimination. Amino acid‐specific signals were identified, especially loss of arginine band. Androgen‐resistant prostate cancer cells presented a higher content of phenylalanine, tyrosine, DNA and Amide III in comparison to PNT2 cells, which possessed greater amounts of L‐arginine and had a B conformation of DNA. The analysis utilized in this study could reliably differentiate the 2 cell lines (sensitivity 95%; specificity 88%).

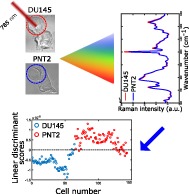

## 1 INTRODUCTION

Androgen‐deprivation therapy is a mainstay management option in men with metastatic or locally advanced prostate cancer. Androgen‐resistance (AR), alternatively known as castrate resistant prostate cancer (CRPC), typically manifests within 12 to 18 months of initiation of treatment, however, it is a major challenge and is responsible for the deaths of 10 000 men each year in the United Kingdom alone. This number is projected to increase and reach over 18.000 deaths in 2035 1. The mechanism of hormonal resistance is complex. Normal prostate epithelial cells require an optimal level of androgens to stimulate growth. Androgen receptor‐mediated signalling, through varied pathways, is the basis for development, differentiation and proliferation of prostate cells. This is what underlies androgen deprivation therapy as a primary treatment for metastatic prostate cancer. Most patients have a favourable response to initial deprivation; however, relapse is very common because of cancer cells developing resistance. Prostate cancer cells circumvent the AR pathway and use other routes, including intracrine androgen production, to proliferate and grow, resulting in upregulation of a number of growth factors, proteins and pathways 2. Analysis of this complex environment is essential to target therapy and in recent years, efforts have been focused on finding novel targeted therapies for CRPC, which is not only chemoresistant but also highly aggressive 3, 4. Amongst a number of optical techniques, Raman spectroscopy is particularly relevant for investigating intracellular biochemical changes 5. Raman spectroscopy detects the vibrational modes of the underlying chemical structure of a cell providing molecular signatures of proteins, lipids and DNA without the need for a cell to be labelled or stained 6.

Raman spectroscopy has been successfully employed in investigating differences between normal and malignant cells in several cancers 7, 8, 9, 10. In prostate cancer, it has been primarily employed coupled with chemometrics as a worthwhile diagnostic technique. Chemometrics, such as principal component analysis (PCA) and linear discriminant analysis (LDA), are powerful tools for analysing Raman spectra of biological samples. Crow et al. successfully used Raman spectroscopy and a PCA/LDA algorithm to discriminate 4 different prostate cancer cell lines (LNCaP, PCa2b, DU145 and PC3) 11. Further Taleb et al. acquired and analysed Raman spectra through different multivariate statistical methods to distinguish between immortalized normal (PNT1A) and metastatic androgen‐dependent malignant (LNCaP) prostate cells 12.

In this paper, we describe the use of Raman spectroscopy to systematically investigate the biochemical differences between immortalized normal prostate cells (PNT2) and metastatic androgen‐independent prostate cancer cells (DU145). PCA of the spectra was initially performed to separate the different cell lines based on their molecular differences, with no apriori assumptions. A subsequent LDA was carried out to determine the components that better align with or discriminate between the 2 cell lines. The discrimination accuracy based on those components was tested by with a k‐fold cross validation. The aim of this work was to gain insights into the biochemical changes in androgen‐independent prostate cancer cells that could aid future research and therapy development.

## 2 METHODS

### 2.1 Cell culture and samples preparation

The immortalized normal human prostate epithelial cell line (PNT2) was purchased from Sigma‐Aldrich, USA and cultured in Roswell Park Memorial Institute medium (RPMI‐1640) containing 10% foetal bovine serum (FBS) and 1% penicillin‐streptomycin. The human prostate androgen‐independent cell line derived from brain metastatic (DU145) was purchased from ATCC, Manassas. and cultured in Dulbecco's Modified Eagle Medium (DMEM) supplemented with 10% FBS and 1% penicillin‐streptomycin. Cells were maintained in the incubator under standard cell culture conditions at 37°C with 5% CO_2_ and 90% Relative Humidity (RH), and sub‐cultured every 2 days to maintain exponential growth. The cell dissociation was processed using 0.05% Trypsin‐Ethylenediaminetetraacetic acid (EDTA). After centrifugation to discard the trypsin supernatant, the cells were seeded (0.8 × 10^6^ seeding density) in 2 different 60‐mm petri dishes, containing each one an autoclaved quartz coverslip (thickness ∼ 0.15 mm). Each petri dish contained the same amount of cells in 4 mL of culture medium. The petri dishes were kept at ambient temperature for 30 minutes to allow the cells to settle down and then incubated for 24 hours at cell culture conditions. Subsequently, the culture medium in the petri dishes was removed and the single monolayer of cells that adhered to the quartz coverslips was washed twice with Phosphate‐buffered saline (PBS) in order to remove the remaining culture medium. At this point, cells were fixed on the slides by using a solution of 70% ethanol in deionised water. The slides were left in the solution for 30 minutes at ambient temperature and then taken out and left to dry, at 90^°^ angle, for other 30 minutes before collecting Raman spectra from the cells.

### 2.2 Raman spectroscopy instrument

**Figure 1:**
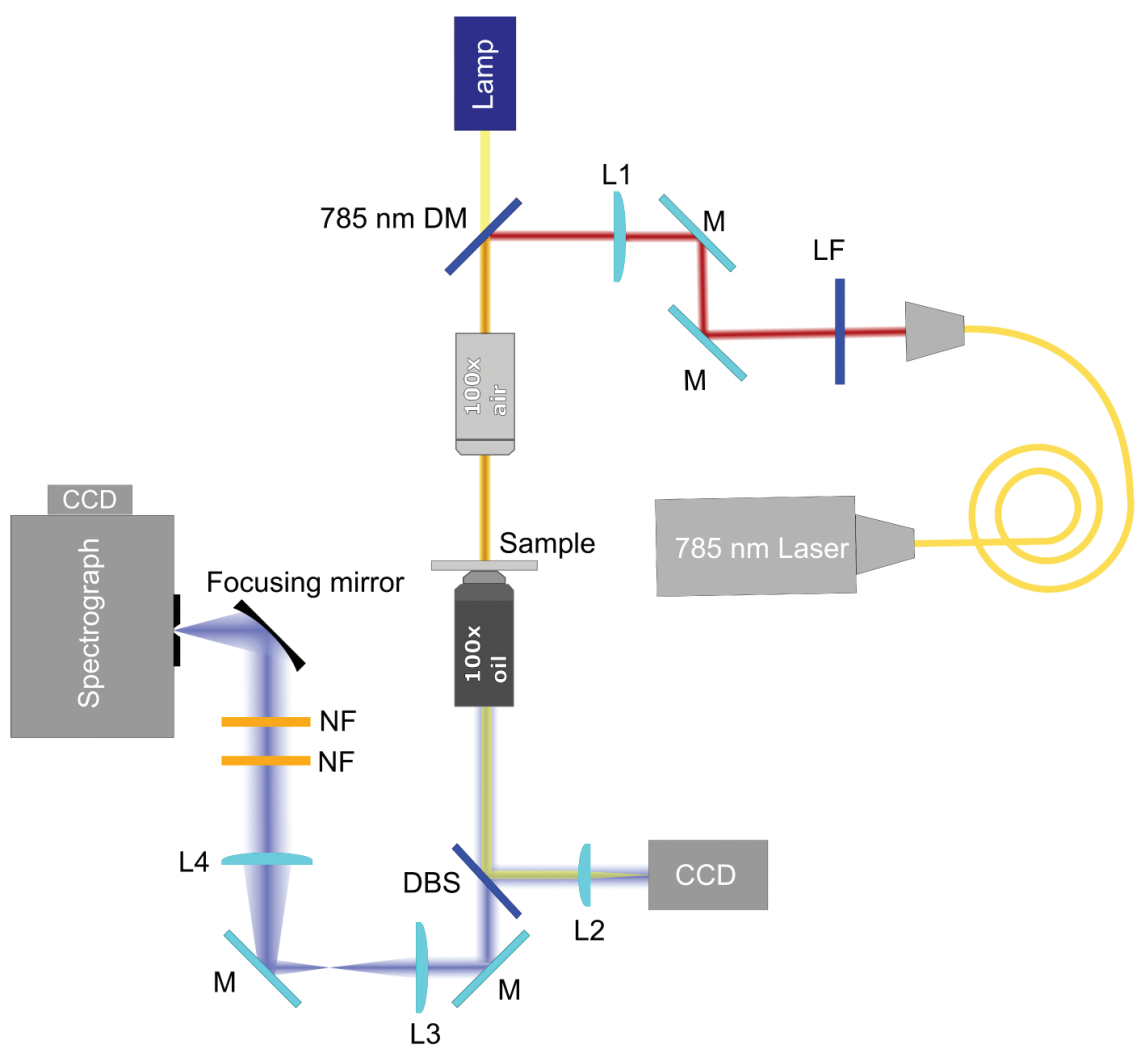
Scheme of the Raman setup used in the study

Raman spectra of single cells were recorded with a custom‐made Raman setup as depicted in Figure 1. A 785‐nm fibre coupled diode laser (Sacher Lasertechnik, Marburg, Germany) was used as excitation source (with power of ∼135 mW on the sample plane). The beam, reflected by a 785‐nm razor edge dichroic mirror (DM), was delivered to the sample through a 100X air microscope objective (Mitutoyo, Japan, G Plan, numerical aperture (NA) = 0.5). A 300‐mm lens (L1) focusing on the back aperture of the objective was employed to modify the initial beam diameter. The beam, having a diameter of ∼2.7 mm at the output of the fibre collimator, was decreased in size in order to be ∼18 μm in diameter on the sample plane. This beam size allowed a single acquisition to represent the spectrum of a single cell. Raman signals from single cells distributed throughout a monolayer were collected with a 100X infinity corrected oil immersion microscope objective (Nikon, Tokyo, Japan, E plan, NA = 1.25) that was focused, after cutting out more than 99.9% of the elastic scattering light through two 785‐nm notch filters (NF), onto the entrance slit of a spectrograph (Andor Shamrock, Andor Technology Ltd., Belfast, UK, entrance slit 200 μm, focal length 500 mm, grating 600 lines/mm). A charge‐coupled device (CCD) camera (Andor Newton DU920P‐BEX2‐DD, Andor Technology Ltd., Belfast, UK) was employed for signal detection. To image the cells, a lamp was switched on before signal collection and a 514‐nm dichroic beamsplitter (DBS) was utilized to reflect a small amount of the visible light towards the CCD camera.

**Figure 2:**
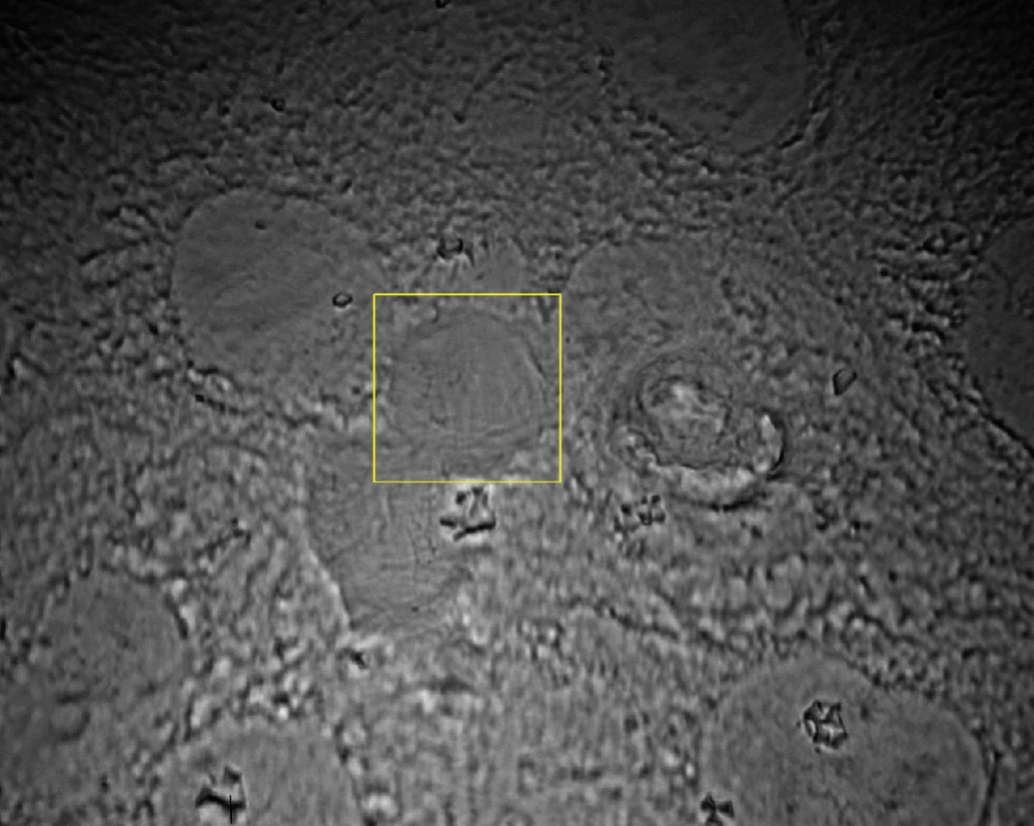
Image of a monolayer of PNT2 cells. Within the yellow box (18 μm in diameter) is the particular cell under investigation

Figure 2 presents the image of a monolayer of PNT2 cells with the cell under investigation highlighted. The lamp was switched off during spectra acquisition. The Raman spectra of 69 individual PNT2 cells and 74 individual DU145 cells were acquired. In order to optimize the Raman signal and reduce the noise, spectra were collected with a 3 seconds exposure time and 20 accumulations. Three different spectral regions were acquired in succession for each cell: the fingerprint region (330–1350 cm^−1^), the bending region (1400–1800 cm^−1^) and the stretching region (2800–3100 cm^−1^).

### 2.3 Spectra analysis

The Raman spectra of cells were analysed by using programmes developed in the Matlab platform (Mathworks, USA). PCA and subsequent LDA were applied to the fingerprint region, from which most of the biochemical differences between the 2 cell lines emanated. PCA was applied to reduce the number of the original variables by projecting them onto a new Cartesian system in which the variables are sorted in descending order of variance. The reduction of the data sets was achieved by retaining only the principal components (PCs) that cumulatively account for more than 95% of the variance in the original data sets. The retained components were utilized as inputs for LDA analysis. While PCA identified the most relevant spectral features of the PCs, allowing the differentiation of cell types to be naturally distinguished on the base of their biomolecular composition, LDA determined the spectral features contained in the linear discriminant function, which maximized the separation of different groups of cells. Internal Matlab routines were applied to pre‐process the spectra prior to PCA/LDA analysis. Each spectrum was processed to remove cosmic rays by applying a Savitzky‐Golay FIR smoothing filter which also elevated the signal‐to‐noise ratio. The baseline arising from the quartz substrate and from biological auto‐fluorescence was also subtracted. Each spectrum was then normalized to the total area under the baseline‐subtracted spectrum to remove a source of variability between the different overall intensities of the Raman features originating from a varying amount of biological material in the sampling volume due to the slightly different sizes and shapes of cells. In order to evaluate the robustness of the PCA/LDA model a 10‐fold cross validation was carried out. For this purpose, the original data were randomly partitioned into 10 equal‐sized subsamples (folds). Each fold was removed from the original data set and the remaining folds imputed in the PCA/LDA analysis to derive the classification model. Eventually, the signal intensities of the spectra in the removed fold were fed into the classification model as blind values in order to determine their classes. This procedure was repeated 10 times (number of folds). The average performance of the PCA/LDA classification models was then obtained.

## 3 RESULTS AND DISCUSSION

**Figure 3:**
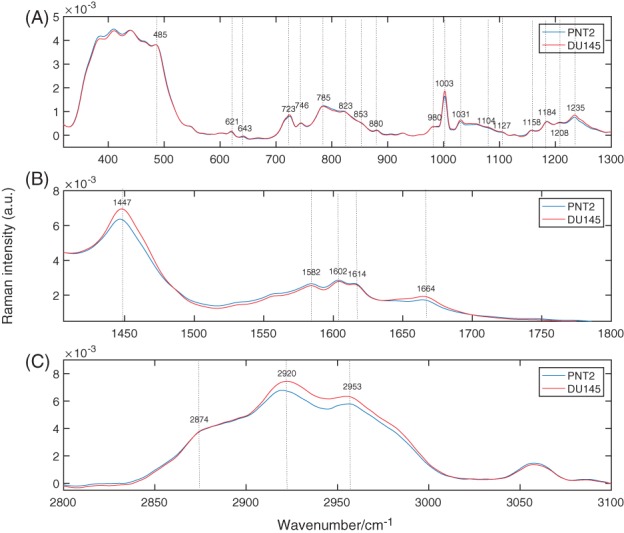
Average Raman spectra of PNT2 and DU145 cells. (A) Fingerprint region, (B) protein region and (C) lipid region

Figure 3 shows the average of the Raman spectra acquired from PNT2 and DU145 cells (A) in the fingerprint region, (B) in the bending region and (C) in the stretching region. Within the fingerprint region, the mean Raman spectrum of PNT2 and DU145 cells (see Figure 3A) features multiple contributions from nucleic acids, proteins and lipids. The nucleic acids bands are due to RNA and DNA bases (adenine [A], thymine [T], guanine [G], cytosine [C] and uracil [U]) and the sugar‐phosphate backbone of DNA. The bands associated with proteins are based on aromatic acids (phenylalanine, tryptophan and tyrosine), amide groups of secondary protein structures and stretching or deformation of CH and CN groups. Certain contributions from lipids are also present. Figure 3B shows the spectral region where the bending vibrational modes of proteins are visible, while Figure 3C depicts the contributions of the CH stretching vibrational modes of lipids and proteins. Bands assignment is summarized in Table 1.

**Table 1:** Raman bands assignment

Band	Assignment	Reference
485	Glycogen	13
497	L‐arginine	14
621	C‐C twisting mode of phenylalanine (proteins)	13
643	C‐C twisting mode of tyrosine	13
723	DNA	8
746	T (ring breathing mode of DNA/RNA bases)	8
785	U,T,C (ring breathing mode of DNA/RNA bases)	8
823	Out‐of‐plane ring breathing, tyrosine (proteins)	15
853	Ring breathing mode of tyrosine (proteins)	13
880	Tryptophan, *δ*(ring) (proteins)	16
980	C‐C stretching *β*‐sheet (proteins)	17
1003	Phenylalanine symmetric ring breathing (proteins)	18
1031	C‐H phenylalanine (proteins)	13
1104	Phenylalanine (proteins)	19
1158	C‐C/C‐N stretching (proteins)	15
1184	Cytosine, guanine, adenine	13
1208	C‐*C*_6_ *H*_6_ phenylalanine (proteins)	19
1235	Amide III (proteins)	19
1447	*CH*_2_ bending mode of proteins (marker for proteins concentration)	19
1582	C = C bending mode of phenylalanine (proteins)	19
1602	C = C bending mode of phenylalanine and tyrosine (proteins)	19
1614	C = C bending mode of tyrosine and tryptophan (proteins)	19
1664	Amide I (proteins)	19
2850–2975	*CH*_2_ symmetric stretching (lipids)	19
2910–2965	*CH*_3_ symmetric and asymmetric stretching (lipids)	19

From Figure 3B it is clear that DU145 cells have higher protein content, as indicated by the band at 1447 cm^−1^, which was identified as a marker of protein concentration (8, 20). The band from 1600 to 1800 cm^−1^ is the Amide I band, mostly attributable to the C = O stretching vibrations of the peptide backbone.

**Figure 4:**
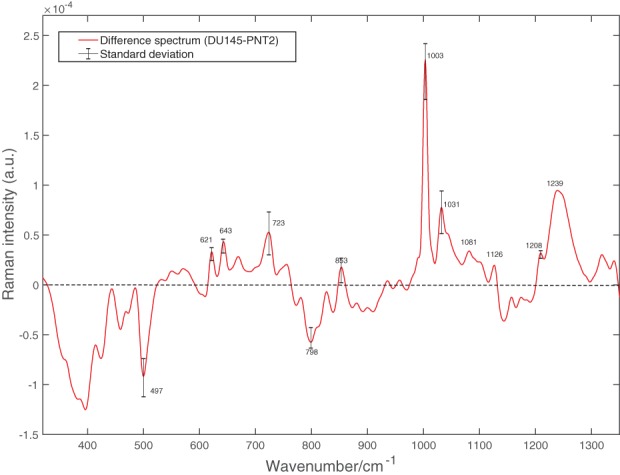
Difference between the mean spectra of DU145 cells and that of PNT2 cells in the fingerprint region shown in Figure 3A. The standard deviation calculated as the difference of the standard deviations for relative amount of biochemical components in DU145 and PNT2 cells is provided at specific wavenumbers of interest

To identify specific constituents, the differences between the 2 cell lines in the fingerprint region had to be considered. Figure 4 shows the difference in spectra between the mean spectrum of DU145 and that of PNT2 cells. Positive bands correspond to compounds present in greater concentration in DU145 cells, while negative bands correspond to compounds more abundant in PNT2 cells. Positive bands are attributable to phenylalanine (621, 1003, 1031 and 1208 cm^−1^) and tyrosine (643 and 853 cm^−1^). Additionally, the band associated with DNA at 723 cm^−1^ is positive, meaning that greater DNA content is present in DU145 cells. The positive band at 1081 cm^−1^ is because of the CN stretching modes of proteins and CC stretching modes of lipids. Meanwhile, the positive band at 1126 cm^−1^ is attributable to the stretching modes of CN featured in proteins and to the vibrational modes of CO present in carbohydrates. The ratio of these 2 bands represents the relative lipid/carbohydrate levels in cells 21.

The band at 497 cm^−1^ corresponds to L‐arginine and the band at 798 is associated with the B conformation of DNA, and both are negative, therefore, related to PNT2 cells 12, 14.

**Figure 5:**
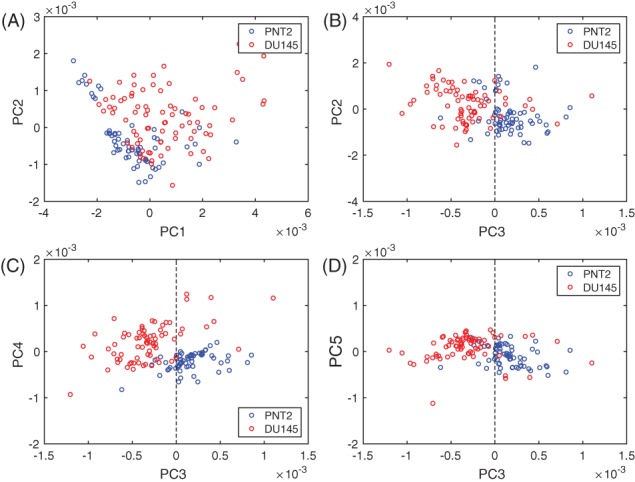
PCA scores plots. (A) Scores generated by PC1 and PC2. (B) Scores generated by PC3 and PC2. (C) Scores generated by PC3 and PC4. (D) Scores generated by PC3 and PC5

**Figure 6:**
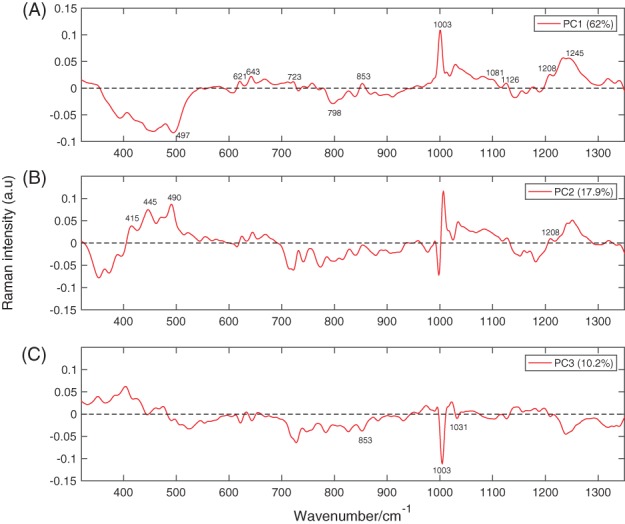
Plots of the first 3 principal components. PC1, PC2 and PC3 account for 62%, 17.9% and 10.2% of the variability in the original data sets, respectively

In order to better capture the biochemical differences between the 2 cell lines, PCA was employed to analyse the spectra in the fingerprint region. It was found that the first 5 PCs accounted for ∼ 97% of the total spectral variability, with the first 3 components accounting for more than 90% of the variability. The plot of the first 3 loadings is presented in Figure 6. The molecular origin of the features in the components can be assigned by comparing the known Raman shifts (Figure 3) with the bands in the loadings. Only the bands clearly arising from the variability in Raman intensity of the bands in the original data sets were assigned. Bands assignment can be found in Table 1. Each loading vector was related to the original spectrum by PC scores, which refer to the weight of that particular biochemical components in each spectrum. In particular, a higher amount of the species giving rise to positive bands in the loading spectrum is present in cells to which higher scores are attributed and vice‐versa: lower amounts of the species producing negative bands can be observed in cells with which lower scores are associated. The score plots shown in Figure 5 highlight the natural groupings of the each of the principal components for the cell types. It is clear that even if the first component allows a grouping to be made, the third component offers better discrimination. The second component, shown in Figure 6B, does not allow any form of discrimination suggesting that it might describe intragroup variability between cells of the same line because of different levels of phosphatidylinositol (band at 415 cm^−1^) in each cell, which is particularly abundant in brain cells, thiocynate (band at 445 cm^−1^) and glycogen (band at 490 cm^−1^).

If the first component in Figure 5 is considered, most of the DU145 cells have positive scores, while the entirety of PNT2 cells have negative scores. This indicates that the species giving rise to positive bands in the spectrum of the loading of the first component (see Figure 6A) are present in higher concentrations in DU145 cells, while the species giving rise to negative bands are found at greater concentrations in PNT2 cells. The spectrum of the first component is very similar to the difference spectrum between the 2 cell lines (see Figure 4), validating what was observed earlier: DU145 cells have more phenylalanine, tyrosine, DNA and Amide III, while PNT2 cells have greater levels of L‐arginine and B conformation DNA.

If the third component is considered, negative scores are associated with DU145 cells, and positive scores with PNT2 cells. Thus, the species with positive bands in the spectrum of the loading of the third component (see Figure 6) should be present in higher concentrations in PNT2, while the species giving rise to negative bands should be more abundant in DU145 cells. This is indeed the case, the content of phenylalanine (1003 and 1031 cm^−1^) and tyrosine (853 cm^−1^) is higher in DU145 cells. Moreover, the third component being that with better grouping between the 2 cell lines suggests that the content of phenylalanine and tyrosine might be a key factor in discriminating between them.

**Figure 7:**
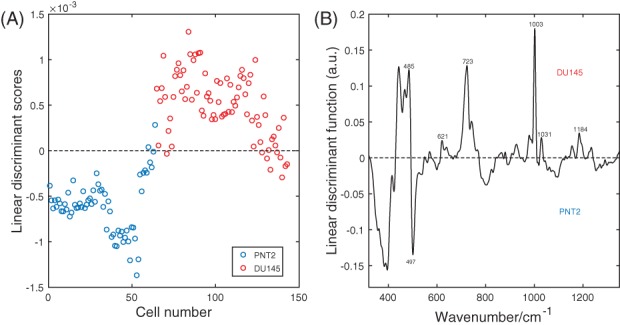
(A) Scatter plot of the linear discriminant scores of PNT2 and DU145 cells spectra using PCA/LDA. (B) Linear discriminant function

In order to verify which biochemical components discriminate the 2 groups of cells, the 5 PCs that described the most of the variance between the spectra were retained for the LDA, while the less significant PCs were discarded. While PCA retained only the PCs that described most of the variance in the data set, LDA generated the linear discriminant function that described the most variance between the 2 different groups of cells. The distribution of LDA scores in Figure 7A clearly reveals that the classification model based on PCA and LDA differentiates PNT2 cells from DU145 cells. Figure 7B features the discriminant function vector along which the 2 cell lines are best separated. In the LDA‐function positive bands are associated with DU145 cells, while negative bands are associated with PNT2 cells.

The bands that are most relevant for the discrimination of the 2 cell lines are those that can be assigned in the LDA‐function spectrum. Features of glycogen at 485 cm^−1^, phenylalanine at 621, 1003 and 1031 cm^−1^, DNA at 723 cm^−1^ and nucleic acids at 1184 cm^−1^ were influential for the assignment into the class DU145. On the other hand, the content of L‐arginine at 497 cm^−1^ led to a classification of PNT2. A 10‐fold cross validation was used to predict the discrimination accuracy based on the biochemical components individuated by the PCA/LDA model as described in the *Spectra Analysis* section. The original data prior to PCA (Raman intensities of the spectra of PNT2 and DU145 cells) were randomly partitioned into 10 equal sized folds. Each fold was employed once as validation data in the PCA/LDA model, while the rest of the folds were utilized as training data. The process was iterated 10 times. The number of correctly identified cases was 132 out of 143 leading to an accuracy of 92.3%. The sensitivity, expressed as the number of correctly identified cancer cells over the total number of DU145 cells was found to be 95%. The specificity, expressed as the number of correctly identified healthy cells over the total number of PNT2 cells was determined to be 88%.

We also conducted a negative control test for eliminating the possibility of spurious correlations driven discrimination 22. The spectra were assigned with different random class labels without taking into account their true class origins. We then repeated a 10‐fold cross validation randomly partitioning the original data set to which random classes are assigned as described above. The process was repeated 100 times with 100 different random class labels vectors. We found an average correct classification rate of 50.8% with standard deviation of 4.9%. The rate of correct classification is relatively low when compared to the 92.3% accuracy obtained with the true class labels and it is consistent with the likelihood of random selection of the true class label, 1/2. This excludes the presence of chance correlations in the PCA/LDA model.

The findings of the present study have implications for future research, including in‐depth mechanistic research into changes metastatic cells acquire to adapt into new environments such as organs. Previous studies conducted by Hedegaard et al. 23 and Winnard Jr et al. 24 using breast cancer isogenic cell lines have shown that this phenomenon is common, however, there are no studies in prostate cancer. Furthermore, with novel chemotherapeutic agents being introduced for the treatment of hormone‐resistant prostate cancer, it is important to develop and validate new technologies (such as Raman spectroscopy) to monitor changes in the phenotype of cells as a marker of response to these treatments.

## 4 CONCLUSIONS

In this study, we used a combination of Raman spectroscopy and PCA/LDA to investigate biochemical changes in androgen‐independent prostate cancer cells (DU145) with respect to normal immortalized prostate cells (PNT2). A PCA analysis was initially carried out on the spectra to allow the 2 cell lines to be naturally discriminated on the basis of their biochemical compositions. DU145 cells had greater phenylalanine, tyrosine, DNA and Amide III vs PNT2 cells, which contained more L‐arginine and B conformation DNA. Thereafter, LDA was conducted on the output of the PCA to determine which biochemical species enable discrimination between the 2 cell lines.

Higher glycogen, phenylalanine, DNA and nucleic acid content were determinants for assigning cells to the DU145 class, while greater levels of L‐arginine was decisive for attributing cells to the PNT2 class.

Pelletier et al. and Pescador et al. demostrated in 2 independent studies that glycogen promotes tumour growth in low oxygen conditions 25, 26. The potential of deprivation of tyrosine and phenylalanine in treating different kind of metastatic cancers, including prostate cancer, has been reported in several studies 27, 28, 29, 30. Ma et al. observed that L‐arginine can block the formation and development of colorectal tumours 31. Abnormalities in arginine metabolism enzymes have been observed in several types of tumours and those cancers support biological processes by relying on extracellular arginine. Therefore, arginine deprivation is currently being investigated as a novel cancer therapy 32, 33. As such, the present work has shown the potential of Raman spectroscopy not only as diagnostic technique, but also to detect biomolecular changes in androgen‐independent prostate cancer cells that could help in establishing novel treatment approaches.

## AUTHOR BIOGRAPHIES

Please see Supporting Information online.

## Supporting information

Author Biographies
