# Supplementary material for: Raman spectroscopy for accurately characterizing biomolecular changes in androgen‐independent prostate cancer cells
Source: J Biophotonics. 2017 Nov 23;11(3):e201700166. doi: 10.1002/jbio.201700166 (PMC6538931; doi:10.1002/jbio.201700166)

**Stella Corsetti** holds a BSc in Biomedical Engineering and a MSc in Bio-Nanotechnology Engineering obtained at the University of Rome 'La Sapienza'. She received her PhD in Chemical Engineering (optics and spectroscopy) from the University of Aberdeen in 2016. She subsequently worked as PDRA at the College of Life Sciences in Dundee where explored the use of Raman spectroscopy and surface enhanced Raman spectroscopy (SERS) in drug discovery and medical diagnostics. She is currently a PDRA at Lancaster University. Her research interests concern with the development and application of optical and spectroscopic techniques for biomedical diagnostics and environmental sensing.

**
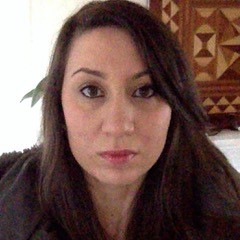
**

**Thomas Rabl** studied physics at the Leopold Franzens Universität in Innsbruck in the Institute for Ion Physics and Applied Physics where he completed his master's degree. Nowadays Thomas is a Marie-Curie fellow in an interdisciplinary PhD program at the University of Dundee. His main interests are equipment development, medical physics, spectroscopy and optics.


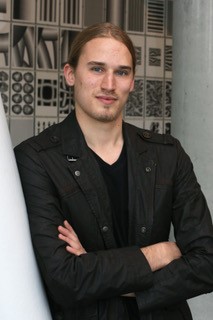


**David McGloin** is a Reader in Physics and Associate Dean for Research in the School of Science and Engineering at the University of Dundee, where he has worked for 10 years. He has a background in atomic and laser physics, with a focus on optical manipulation techniques. His current research interests focus on the development and application of tools in photonics and microfluidics for use in biological and medical science. These include optical trapping and hydrodynamic trapping, along with Raman Spectroscopy and super-resolution microscopy.

**
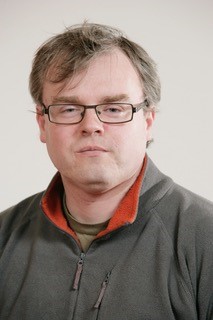
**

**Ghulam Nabi** was a clinical academic in urology trained in Surgery and Urology in India, East of Scotland Training Programme in Urology, France and Germany. Previously Clinical Lecturer at the University of Aberdeen, Professor Nabi was appointed to Chair in October 2015. Besides heading the academic section of Urology at the University of Dundee, Professor Nabi is lead for prostate cancer Surgery in Tayside and chair of Tayside Urological Cancers Network. He introduced a number of minimally invasive surgical techniques for Tayside including laparoscopic surgery in prostate cancer, single port surgery and cryoablation for renal cancers. His research focuses on “early detection and minimally invasive treatment” for urological cancers.


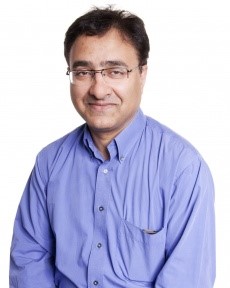

Supplement: Supplementary file 1 — Author Biographies [file JBIO-11-na-s001.doc]
